# One Heresy and One Orthodoxy: On Dialetheism, Dimathematism, and the Non-normativity of Logic

**DOI:** 10.1007/s10670-022-00528-8

**Published:** 2022-03-09

**Authors:** Heinrich Wansing

**Affiliations:** Department of Philosophy I, Ruhr University Bochum, Universitätsstraße 150, 44780 Bochum, Germany

## Abstract

In this paper, Graham Priest’s understanding of dialetheism, the view that there exist true contradictions, is discussed, and various kinds of metaphysical dialetheism are distinguished between. An alternative to dialetheism is presented, namely a thesis called ‘dimathematism’. It is pointed out that dimathematism enables one to escape a slippery slope argument for dialetheism that has been put forward by Priest. Moreover, dimathematism is presented as a thesis that is helpful in rejecting the claim that logic is a normative discipline.

## Introduction

Graham Priest is known for defending three unorthodox theses in logic and metaphysics, which he refers to as “three heresies” (Priest, 2013):

1. Dialetheism, the thesis that some contradictory sentences are true,
2. Noneism, the thesis that some objects do not exist, and
3. The thesis that the relation of numerical identity is non-transitive.^1^

Although the three heresies form a closely interrelated part of Priest’s thinking, the focus of the present paper is only on the first heresy, dialetheism.^2^ It will be scrutinized, an alternative to it will be presented, and the attention will then be directed to a certain orthodoxy in philosophical logic, namely the view that logic is a normative discipline. As to Priest’s first heresy, I will consider a slippery slope argument in favour of dialetheism put forward by Priest in (2000). My response to the slippery slope argument is to deflect the slope by strictly separating logic from metaphysics and by conceiving of logic as the discipline that studies the most general laws of *information flow*. Priest would agree that logic is about what follows from what, so the task is to detach logical consequence from truth and falsity as metaphysical notions.

Although Priest appears to be a die-hard heretic, he endorses the quite orthodox view that logic is a normative discipline. I do not think of logic as being normative, and in expounding that position, I will consider an objection to an entanglement of logic with the normative put forward by Shramko (2014) and take up a footnote to another objection presented by Russell (2020).

The paper is organized as follows. In Sect. 2, I will present dialetheism, and I will draw a distinction between different kinds of metaphysical dialetheism. Next, in Sect. 3, I will introduce an alternative to dialetheism, namely what I will call ‘dimathematism’. Whilst dialetheism is a metaphysical view, dimathematism is an informational thesis. Section 4 is then dealing with Priest’s slippery slope towards dialetheism, and I will point out that the slippery slope can be deflected towards dimathematism. Eventually, in Sect. 5, I will address the orthodox thesis, subscribed to by Priest, that logic is a normative discipline. I will argue that dimathematism helps dissociate logic from normativity.

## Dialetheism

### What is Graham Priest’s Dialetheism?

Priest (2013, p. 10) [see also Priest (2014, p. xviii)] presents his first heresy as a *metaphysical* view [notation slightly adjusted]:

Dialetheism is a metaphysical view: some contradictions are true. That is, where $\sim$ is negation, there are sentences, propositions (or whatever one takes truth-bearers to be), *A*, such that both *A* and $\sim\!{A}$ are true. Given that *A* is false iff its negation is true, this is to say that there are some *A*s which are both true and false.

It is generally agreed that “[i]t is not easy to say what metaphysics is,” van Inwagen and Sullivan (2020). The Metaphysical Society of America^3^ describes its purpose as “the study of reality,” and since Priest in (2014) uses the notion of *metaphysical reality*, it may suffice here to characterize metaphysics as the study of a mind-independent reality, see also Fine (2001). His presentation of dialetheism as a metaphysical view makes it clear that he considers truth and falsity as metaphysical notions, so that if a contradiction is true, it is actually true, true in reality. Note, however, that according to Priest (2005) *all* major theories of truth are compatible with dialetheism, including the deflationist theory of truth, which in its different varieties is usually seen as dissociating the notion of truth from metaphysics. In the brief discussion about dialetheism, realism, and anti-realism in Priest et al. (2018, Sect. 6), it is highlighted that there are anti-realist dialetheic theories of truth such as Beall’s (2004) *constructive methodological deflationism*; see also Kroon’s (2004) *fictionalist dialetheism*.

Mares (2004) introduces the notion of *semantic dialetheism* and explains the difference between metaphysical and semantic dialetheism as follows (Mares, 2004, p. 270): “The metaphysical dialetheist holds that there are aspects of the world (or of some possible world) for which any accurate description will contain a true contradiction. Semantic dialetheism, on the other hand, maintains that it is always possible to redescribe this aspect of the world, using a different vocabulary (or perhaps vocabularies), consistently without sacrificing accuracy.” In a discussion of semantic dialetheism as suggested by Mares, Priest (2006, pp. 299–302) eschews a confession to dialetheism as a metaphysical theory and explains that although Mares takes him to be a metaphysical dialetheist, “there may well be no uniform answer to the issue of metaphysical dialetheism.” However, Priest grants that dialetheism implies that “the world is contradictory.” Priest (2018, Footnote 9), remarks with respect to metaphysical dialetheism that “[a] number of people have taken me (mistakenly) to be committed to this kind of dialetheism,” the latter being the thesis that “that there may be a *more profound* sort of dialetheia, a contradiction in the world itself, independent of any linguistic/conceptual considerations” (my emphasis) (Priest, 2018, p. 5).

More recently, Priest (2019, p. 59) discusses what he calls *psychological dialetheias*:

By a psychological dialetheia I mean a dialetheia which describes some agent’s mental state. Normally, our mental states are, it would seem, quite consistent. If I am thinking of the Taj Mahal, I am not also not thinking of it. If I see a polar bear, I do not also not see it. But one should not be taken in by an inadequate diet of examples|as Wittgenstein put it. Unusual things may happen in unusual situations; and arguably some odd sorts of situations may give rise to psychological dialetheias.

In this paper I will consider Priest’s dialetheism as a metaphysical theory. The focus on this kind of dialetheism is justified by the fact that it is the most widely discussed and influential version of dialetheism. It should be clear that the above basic characterization of metaphysical dialetheism requires some qualification. A sentence is, by definition, a sentence in a given language. Even if reference to a language is suppressed, nevertheless dialetheism is to be understood as saying that there is a language, *L*, and there are sentences *A* from *L*, *L*-sentences, such that *A* is both true and false. If *L* is a formal language, its sentences are formulas (or closed formulas if *L* contains variable-binding operators). Moreover, if an *L*-sentence *A* is true or false, then it is not just true or false but true or false in a situation where *A* qualifies for receiving a semantical value. Representations of such situations are usually called *L*-models, *L*-interpretations, or *L*-structures, and *L*-sentence are evaluated as true or false in *L*-models.

In the following, I will to a large extent suppress reference to languages. The tacit existential quantification over languages will, however, become relevant when I will address the question whether or not dialetheism or dimathematism are trivial theories. The relativization to models is essential because there are languages *L*, *L*-sentences *A*, and *L*-models ${\mathfrak {M}}$, ${\mathfrak {M}}'$ such that *A* is true in ${\mathfrak {M}}$ but false in ${\mathfrak {M}}'$. Also, the semantic evaluation relation may depend on additional parameters, in particular, if the language is a modal language, semantic evaluation may depend on possible worlds or states. An *L*-sentence *A* is then evaluated at a world from an *L*-model, and *A* is said to be true in an *L*-model ${\mathfrak {M}}$ iff *A* is true at every world from ${\mathfrak {M}}$’s non-empty set of worlds.

Both metaphysical dialetheism and the doctrine to be developed, dimathematism, rely on the notion of a model, and therefore it is appropriate to clarify the use of that notion for present purposes.^4^ Whilst model-theoretic semantics is usually associated with realism, anti-realism is often associated with proof-theoretic semantics. In what follows, a model or situation is understood as an abstract mathematical structure that stands in a relation to something of which it is a model, without thereby necessarily creating a metaphysical commitment. The models are not assumed to represent any kind of *reality* or *way the world really is*. In Sect. 3, a distinction will be drawn between truth (falsity) at a state in a model and support of truth (falsity) at a state from a model. With that contrast explicitly made, truth at a state from a model may be seen as a metaphysical concept. If a statement then is true at a state, what the statement says *is the case at that state*. If a statement is false at a state, what the negation of the statement says *is the case at that state*. Support of truth (falsity), however, does not imply truth (falsity) in a metaphysical sense, that is, does not imply real truth (real falsity).

For our presentation of dialetheism we have to consider some other, more specific setups as well. If every model ${\mathfrak {M}}$ is equipped with a unique designated possible world, then typically an *L*-sentence *A* is said to be true in an *L*-model ${\mathfrak {M}}$ iff *A* is true at the designated world from ${\mathfrak {M}}$. Also, sometimes the set of worlds is bi-partitioned into a set of normal and non-normal worlds (often referred to as the possible and the impossible worlds), for a survey see Berto and Jago (2018). Possible worlds are usually assumed to be complete and consistent states, i.e., for every *L*-formula *A* and every possible world *w* from an *L*-model ${\mathfrak {M}}$, *A* is either true or false at *w*, but not both. If this restriction is relaxed, it is not uncommon to talk about situations instead of worlds. Moreover, if a distinction is drawn between normal and non-normal worlds, then it is not unusual to define truth in an *L*-model ${\mathfrak {M}}$ not as truth at *every* world from ${\mathfrak {M}}$ but as truth at *every normal* world from ${\mathfrak {M}}$.

Like truth in a model, semantic consequence understood as truth preservation may thus be defined with respect to different subsets of the set of states from a model. If ${\mathfrak {C}}$ is the non-empty class of all models under consideration and $\varGamma \cup \{A\}$ is a set of sentences, then semantic consequence (entailment) is often defined in one out of three ways, namely: $\varGamma$ entails *A* ($\varGamma \models A$) iff

($$\models _a$$)
for every model ${\mathfrak {M}} \in {\mathfrak {C}}$ and every world *w* from ${\mathfrak {M}}$, if *B* is true at *w* for every $B \in \varGamma$, then *A* is true at *w*, or
($$\models _b$$)
for every model ${\mathfrak {M}} \in {\mathfrak {C}}$, if *B* is true at ${{\mathbf {d}}}_{{\mathfrak {M}}}$ for every $B \in \varGamma$, then *A* is true at ${{\mathbf {d}}}_{{\mathfrak {M}}}$, where ${{\mathbf {d}}}_{{\mathfrak {M}}}$ is the unique distinguished state from ${\mathfrak {M}}$, or
($$\models _c$$)
for every model ${\mathfrak {M}} \in {\mathfrak {C}}$ and every normal world *w* from ${\mathfrak {M}}$, if *B* is true at *w* for every $B \in \varGamma$, then *A* is true at *w*.

It seems fair to say that ($\models _a$), defining entailment as truth preservation at every world, is the prevailing approach. If the set of states of a model is bi-partitioned into a set of possible worlds and a set of impossible worlds *and* at the same time every model is equipped with a unique designated state, then the motivation for a choice between ($\models _b$) and ($\models _c$) is maybe not so clear. In any case one would have to say whether ${{\mathbf {d}}}_{{\mathfrak {M}}}$ is normal for every ${\mathfrak {M}}$, or whether ${{\mathbf {d}}}_{{\mathfrak {M}}}$ is non-normal for every ${\mathfrak {M}}$, or whether it is allowed that for some models ${\mathfrak {M}}$, ${{\mathbf {d}}}_{{\mathfrak {M}}}$ is normal, and for some models ${\mathfrak {M}}'$, ${{\mathbf {d}}}_{{\mathfrak {M}} '}$ is non-normal.

Priest draws a distinction between truth or falsity simpliciter and truth or falsity in an interpretation.^5^ Priest (1999, Footnote 13) explains that

*being true in* is quite distinct from *being true (simpliciter)* $\ldots$. The latter notion is a property, or at least, a monadic predicate, and has nothing, in general, to do with sets. One might be interested in it for all kinds of reasons, which it is unnecessary to labour. The second is a relation, and a set theoretic one, at that; and the only reason that one might be interested in it is that it is a notion necessary for framing an account of validity. The two notions are not, of course, entirely unrelated. One reason we are interested in valid inferences is that we can depend on them to preserve truth, actual truth. Hence it is a desideratum of the notion of truth-in-a-structure that there be a structure, call it the actual structure, such that truth (period) coincides with truth in it. The result is then guaranteed. No doubt this imposes constraints on what one’s account of structure should be, and on how the truth-in relation should behave at the actual structure. But one should not suppose that just because the actual structure possesses certain features (such as, for example, consistency or completeness), other structures must share those features: we reason about many things other than actuality. Similarly, recursive truth conditions may collapse to a particularly simple form at the actual structure because of certain privileged properties. But that is no reason to think that they must so collapse at all structures.

Priest’s truth simpliciter and falsity simpliciter are thus truth, respectively falsity, in a distinguished model, *the actual structure*. Priest (2002, p. 656 f.) relativizes the semantic evaluation of sentences to possible worlds, considers *the actual world*, and presents dialetheism as a thesis concerning the actual world:

Someone may well hold that there are possible worlds that are inconsistent without holding that the actual world is. After all, the actual world is special. Truth at that world coincides with truth simpliciter. And truth has special properties all of its own. For example, one might well hold that for any *A*, $\sim\!{A}$ is true iff *A* fails to be true, whilst this is not true of worlds in general. The claim that the actual world is inconsistent, though, is dialetheism.

In *One*, (Priest, 2014, p. *xxii*, f.), Priest writes:

Worlds are many. Some of them are possible; some of them are impossible. The actual world, @, is one of the possible ones: 
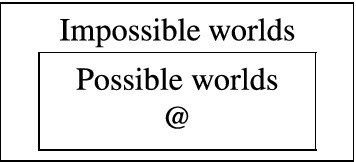

Given dialetheism, there are contradictions true at the actual world. One might wonder, therefore, what makes a world impossible. Answer: an impossible world is one where the laws of logic are different from those of the actual world (in the way that a physically impossible world is a world where the laws of physics are different from those of the actual world). Given the plurality of worlds, truth, truth conditions, and so on, must be relativized to each of these. That is a relatively routine matter.

What is not an entirely routine matter, perhaps, is a choice between the above options ($\models _b$) and ($\models _c$) for defining semantic consequence. At any rate, since for Priest the designated state, @, is a possible world, being possible for a world *w* does *not* exclude being inconsistent in the sense that some contradiction $A \wedge \sim\!{A}$ is true at *w*.^6^

### Single Model Dialetheism and Multiple Models Dialetheism

If we assume that there is a unique actual world, @, and that it belongs to the unique actual structure, ${\mathfrak {A}}$, then dialetheism can be presented as what may be called *single model* *dialetheism*:

*Single model dialetheism* is a metaphysical view: some contradictions are true at the unique actual world, @, in the unique actual model, ${\mathfrak {A}}$. That is, where $\sim$ is negation, there are sentences, propositions (or whatever one takes truth-bearers to be), *A*, such that both *A* and $\sim\!{A}$ are true at @ in ${\mathfrak {A}}$. Given that *A* is false iff its negation is true, this is to say that there are *A*s which are both true and false at @ in ${\mathfrak {A}}$.

Single model dialetheism need not deny that there is more than one model, but the unique actual world belongs to exactly one model, the actual interpretation. The set of worlds of the actual model need not be a singleton, and there may be other models with their sets of worlds, but these sets do not contain @.

In the semantics of quantified modal logic one usually admits trans-world identity of individuals: One and the same individual can exist in more than one possible world. Similarly, one might assume trans-model identity of possible words, in particular, one might assume that the unique actual world, @, may belong to the set of worlds of more than just one possible worlds model. One could then come up with what may be called *multiple models dialetheism*:

*Multiple models dialetheism* is a metaphysical view: some contradictions are true at the unique actual world, @, in some model ${\mathfrak {M}}$. That is, where $\sim$ is negation, there are sentences, propositions (or whatever one takes truth-bearers to be), *A*, and models, ${\mathfrak {M}}$, such that both *A* and $\sim\!{A}$ are true at @ in ${\mathfrak {M}}$. Given that *A* is false iff its negation is true, this is to say that there are some models ${\mathfrak {M}}$ and *A*s which are both true and false at @ in ${\mathfrak {M}}$.

Models to which the unique actual world belongs may be called ‘actual models’ or ‘actual interpretations.’ Priest (2013, p. 12, 2014, p. *xx*) explains that “Dialetheism is, however, the view that some *actual situations* are such that, in them, ${\mathfrak {T}}$ and ${\mathfrak {F}}$ have a proper overlap,” (my emphasis) where ${\mathfrak {T}}$ is the class of truth-bearers true in a given situation (interpretation) and ${\mathfrak {F}}$ is the class of truth-bearers false in a given interpretation, so that ${\mathfrak {F}}$ is a class of falsity-bearers. Priest (2000, p. 230) explains that a model may be thought of as representing a possible situation. An actual model could then be understood as representing an actual situation comprising @ (or what is represented by @).

However, one may also assume that every possible worlds model ${\mathfrak {M}}$ has its own unique actual world @_M_. In Nelson and Zalta (2012) for instance, a model for the modal propositional logic **S5** is introduced as a triple ${\mathfrak {M}}$ $=$ $\langle W, @_{{\mathfrak {M}}},V\rangle$ (notation modified), where *W* is a non-empty set of possible worlds, @_M_ $\in$ *W*, and *V* is a valuation function that maps each atomic sentence *p* of the language of **S5** to a subset of *W*, intuitively to the set of possible worlds where *p* is true in ${\mathfrak {M}}$. Truth in such a model ${\mathfrak {M}}$ is then defined in Nelson and Zalta (2012) as truth at @_M_ in ${\mathfrak {M}}$. As an instance of ($\models _b)$-type entailment, the notion of real-world validity, is defined as validity in every model $\langle W, @_{{\mathfrak {M}}},V\rangle$. Likewise, Priest in (2006, p. 85) introduces interpretations $\langle W, R, G, v\rangle$ where *W* is a non-empty set of worlds, *R* is a binary accessibility relation on *W*, *G* is a distinguished element of *W*, “the “real world” or assignment which is in accord with the actual,” and *v* is a valuation function mapping pairs of worlds and atomic sentences to a three-element set of semantical values. Semantical consequence is of type ($\models _b$) and is defined as truth preservation at *G* for every model $\langle W, R, G, v\rangle$.

### Dialetheism and Strong Dialetheism

The generality of the latter semantics and its presentation by one of the founders of dialetheism speaks in favour of eliminating not only single model dialetheism but also multiple models dialetheism from further consideration.^7^ Irrespective of a choice between ($\models _a$), ($\models _b$), and ($\models _c$), a semantics where every possible worlds model ${\mathfrak {M}}$ comes with its unique actual world @_M_ gives rise to what I will refer to as *dialetheism*:

*Dialetheism* is a metaphysical view: some contradictions are true in some model ${\mathfrak {M}}$ at the actual world @_M_ from ${\mathfrak {M}}$. That is, where $\sim$ is negation, there are sentences, propositions (or whatever one takes truth-bearers to be), *A*, and models, ${\mathfrak {M}}$, such that both *A* and $\sim\!{A}$ are true at @_M_ in ${\mathfrak {M}}$. Given that *A* is false iff its negation is true, this is to say that there are some models ${\mathfrak {M}}$ and *A*s which are both true and false at @_M_ in ${\mathfrak {M}}$.

If *L* is the language under consideration, dialetheism thus holds hat there are *L*-models ${\mathfrak {M}}$ and *L*-formulas *A* which are both true and false at @_M_ in ${\mathfrak {M}}$. Once we consider dialetheism so understood, it is natural to consider the non-empty class ${\mathfrak {C}}$ of all *L*-models and to replace the particular quantification over models by universal quantification, thereby obtaining what may be called *strong dialetheism*:

*Strong dialetheism* is a metaphysical view: some contradictions are true in every model ${\mathfrak {M}}$ at the actual world @_M_ from ${\mathfrak {M}}$. That is, where $\sim$ is negation, there are sentences, propositions (or whatever one takes truth-bearers to be), *A*, such that for every model ${\mathfrak {M}}$, both *A* and $\sim A$ are true at @_M_ in ${\mathfrak {M}}$. Given that *A* is false iff its negation is true, this is to say that there are *A*s which are both true and false at @_M_ in every model ${\mathfrak {M}}$.

If models ${\mathfrak {M}}$ come with their own unique actual world @_M_, obviously strong dialetheism implies dialetheism. One may wonder whether Priest is an advocate of dialetheism as just presented (or even of strong dialetheism). Maybe that is not so clear. Priest (2000, p. 228) explains that “the thought that some inconsistent but non-trivial theories may be *true* $\ldots$ is a view now called ‘dialetheism’.” This is the third level of paraconsistency listed in Priest (2000), to which a forth level is added in Beall and Restall (2006), Priest et al. (2018), namely dialetheic paraconsistency, the view that “some inconsistent but non-trivial theories are true.”

If “may be *true*” expresses particular quantification over models in the metalanguage, then what we obtain is just dialetheism and this might explain why Priest (2000) refers to the third level of paraconsistency as dialetheism.^8^ Beall and Restall (2006, p. 80) seem to think of the ‘may’ in “may be *true*” as a possibility operator in the object language because they consider an endorsement of $\Diamond (A \wedge \sim\!{A})$ for some *A*. They point out that in a modal expansions of Priest’s (or Asenjo and Priest’s) Logic of Paradox, **LP**, with necessity, $\Box$, and possibility, $\Diamond$, being interdefinable and the necessitation rule being valid, $\sim\Diamond (A \wedge \sim A)$ is provable for any formula *A*, so that by rejecting certain features of **LP**, a coincidence between the third and the fourth level of paraconsistency can be avoided.

However, there is more than one way of representing the claim that some inconsistent but non-trivial theories may be true as a statement in the object language. According to one reading different from Beall and Restall’s, the third level of paraconsistency maintains that there are contradictions that are possibly *true simpliciter*; according to another, also different from Beall and Restall’s, the claim is that there are contradictions that are *possibly true* simpliciter. If, abusing notation because contexts will disambiguate, we employ ‘@’ not just as a name of a possible world but also as a unary connective, the difference it between

(i) $$\Diamond @ (A \wedge \sim\!{A})$$ and
(ii) $$@ \Diamond (A \wedge \sim\!{A})$$

where @*A* is true at a world from a model ${{\mathfrak {M}}}$ iff *A* is true at @_M_. Assuming that possibility is possibility in the modal logic **S5**, then a sentence *A*, in particular a contradiction $(A \wedge \sim\!{A})$, is actually true just in case it is possibly actually true, (i). The direction from left to right holds by the assumption that we are working in **S5**, and if *A* is possibly actually true at some world from a model ${{\mathfrak {M}}}$, then there is a world at which *A* is actually true, but this can only be @_M_. This again might explain why Priest in (2000) refers to the third level of paraconsistency as dialetheism, but we are also left with the second reading, (ii), as giving rise to a weak sort of dialetheism:

**Weak dialetheism** is a metaphysical view: some contradictions are true in some model ${\mathfrak {M}}$ at some possible or logically impossible world accessibly from from $@_{\mathfrak {M}}$. That is, where $\sim$ is negation, there are sentences, propositions (or whatever one takes truth-bearers to be), *A*, and models ${\mathfrak {M}}$, such that both *A* and $\sim\!{A}$ are true at some world accessible from $@_{\mathfrak {M}}$. Given that *A* is false iff its negation is true, this is to say that there are some models ${\mathfrak {M}}$ and *A*s which are both true and false at some world accessible from $@_{\mathfrak {M}}$.

We may assume that dialetheists want dialetheism to be a non-trivial theory: Some sentences are true and false simpliciter but not every sentence is a dialetheia. The non-triviality of dialetheism hardly seems to be in need of justification, but note that there is also trivialism, the claim that every proposition is true (Azzouni, 2007; Kabay, 2010). Although Priest’s favourite paraconsistent logic, **LP**, has a trivial model in which every formula receives the designated value “both true and false”, why should one assume that if there is an actual structure, this structure is represented by the trivial **LP** model? Also, axiomatic extensions of **KD**_LP_, the normal modal logic **KD** based on **LP**, have trivial models in which every formula has the value “both true and false” at every world, but why should one assume that if there is an actual world, this world is represented by a trivial world from a trivial model?

In order to allow for contradictory but non-trivial theories, dialetheists use a paraconsistent logic, and since dialetheism is defined in terms of contradictions understood as sentences of the form $(A \wedge \sim\!{A})$, they have to assume what is called “strong paraconsistency” and to deny *ex contradictione quodlibet* in the form $(A \wedge \sim\!{A}) \models B$, that is, a contradiction $(A \wedge \sim A)$ does not entail any sentence *B* whatsoever.^9^

Moreover, dialetheists substantiate the claim that the class of dialetheias is non-empty not by a reductio ad absurdum but constructively by providing examples. These example have different logical status. Some are sentences such that if they are true in some model, they are clearly false in others. If, for instance, a person *a* is entering the Empire State Building, Graham Priest would claim that there is a state at which the contradiction ‘*a* is in the Empire State Building and *a* is not in the Empire State Building’ is true. But it is certainly false at some state that ‘*a* is in the Empire State Building and *a* is not in the Empire State Building,’ for example at a state where *a* is not in New York. Other examples are provided by legislation inasmuch as some laws happen to be inconsistent theories. As legislation changes, inconsistent laws come and go.

It is usually granted that the most pressing motivation for dialetheism comes with the paradoxes of self-reference, which are maintained to involve necessary dialetheias. Priest (2002, p. 657) explains that

the contradictions involved in the paradoxes of self-reference are, in a sense, inherent in thought $\ldots$ [and] being inherent in thought, are necessarily true

and Priest et al. (2018) explain that “such paradoxes as the Liar provide some evidence for the dialetheist’s claim that some contradictions are *provably* true, in the sense that they are entailed by plain facts concerning natural language and our thought processes.” One may, of course, here think of *provability in a theory*, such as provability in naive set theory or naive truth theory, and one may think of necessity as a kind of necessity different from logical necessity.

*Logically* provable dialetheias should be true in any model whatsoever. If semantic consequence is of type ($\models _a$), logically provable dialetheias should be true at any state, for entailment of type ($\models _b)$, they should be true at every designated state **d**_M_, and for semantic consequence of type ($\models _c$) logically provable dialetheias should be true at every normal world. For a strongly paraconsistent logic the dialetheists are in need of, this means that this logic should be a non-trivial but inconsistent logic. The Logic of Paradox, **LP**, is, however, a non-trivial but non-inconsistent logic.

As a result, we may conclude that if we assume the semantics used in Priest’s (2006), then we obtain dialetheism with respect to certain classes of interpretations, namely the classes of models of certain non-trivial inconsistent theories. We shall now introduce an alternative to dialetheism.

## Dimathematism

### Logic as the Study of Information Flow

According to Frege (1990, p. 39), logic is the science of the most general laws of being true (“die Wissenschaft der allgemeinsten Gesetze des Wahrseins”). Assuming bivalence, logic is then also the science of the most general laws of not being false. Dimathematism, however, conceives of logic as the science of the most general laws of information flow.

In the semantics of the basic paraconsistent logic **FDE**, first-degree entailment logic, it is possible to replace the classical truth values, *true* and *false,* and their metaphysical understanding by four semantical values, T, F, N, and B, which have an informational reading. This informational understanding is essentially Nuel Belnap’s interpretation of T, F, N, and B as ‘told’ values that can be provided to human or artificial information processors concerning some given atomic formula (Belnap 1977a, b):

- N: neither told *true* nor told *false*
- F: told only *false*
- T: told only *true*
- B: both told *true* and told *false*.

**Fig. 1:**
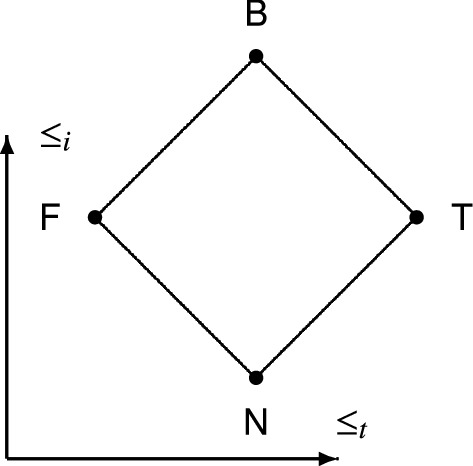
The bilattice *FOUR*_2_

Whilst the told values are provided for atomic sentences by some information sources, their assignment to compound sentences is defined recursively. The values N, F, T, and B can be represented as the elements of the powerset $\mathcal{P}$($\{$*true*, *false*$\}$) of the set of classical truth values: N = $\varnothing$, T = $\{$*true*$\}$, F = $\{$*false*$\}$, and B = $\{$*true*, *false*$\}$, see Dunn (1976, 2000). Under this representation, set-inclusion is a natural information order, $\le _i$, on the set **4** = $\{$ N, F, T, B $\}$. The more elements one of the four truth values contains, the more informative it is. Sometimes or maybe even often, another partial order on **4** is considered to be a truth ordering, $\le _t$, thereby giving rise to the bilattice *FOUR*_2_ in Fig. 1.^10^

If a state *w* from a possible words model ${\mathfrak {M}}$ is such that at *w* an atomic sentence *A* is told only *true*, then the model ${\mathfrak {M}}$ supports the truth of *A* at *w* (or *w* supports the truth of *A* in ${\mathfrak {M}}$) , and if *w* and ${\mathfrak {M}}$ are such that at *w* the sentence *A* is told only *false*, then the model ${\mathfrak {M}}$ supports the falsity of *A* at *w* (or *w* supports the falsity of *A* in ${\mathfrak {M}}$). There is nothing that prevents an atomic formula *A* from possibly being both told *true* and told *false* (by different sources or one and the same source) so that a model may support both the truth and the falsity of *A* at a state *w*, and there is also nothing that prevents *A* from possibly being neither told *true* nor told *false* (by any source) so that a model may neither support the truth nor support the falsity of *A* at a state *w*. In particular, the states may be seen as information states.

Dimathematism takes an informational stance and does not assume a notion of truth simpliciter. It does not assume a unique actual possible world or several actual possible worlds, nor does it assume the actual model or several actual models. Instead it is a thesis about support of truth and support of falsity. The notion of semantical consequence behind dimathematism is that of entailment as information flow, where support of truth is on a par with support of falsity, or if not, this is just to tie up with a traditional bias or because one notion is definable in terms of the other, and vice versa. In the relational semantics for David Nelson’s constructive paraconsistent logic **N4** (Almukdad & Nelson, 1984; Odintsov, 2008), for example, a state *w* supports the falsity of a formula *A* iff *w* supports the truth of $\sim\!{A}$, the strong negation of *A*.

If we want to parallel the distinction between the types of entailment of a single formula *A* from a premise set $\varGamma$ (($\models _a$), ($\models _b$), ($\models _c$)), what we obtain are the following pairs of options, where again ${\mathfrak {C}}$ is the non-empty class of all models under consideration:

($$\models ^{+}_a$$)
for every model ${\mathfrak {M}} \in {\mathfrak {C}}$ and every world *w* from ${\mathfrak {M}}$, if *w* supports the truth of every $B \in \varGamma$, then *w* supports the truth of *A*, and
($$\models ^{-}_a$$)
for every model ${\mathfrak {M}} \in {\mathfrak {C}}$ and every world *w* from ${\mathfrak {M}}$, if *w* supports the falsity of every $B \in \varGamma$, then *w* supports the falsity of *A*, or
($$\models ^{+}_b$$)
for every model ${\mathfrak {M}} \in {\mathfrak {C}}$, if ${{\mathbf {d}}}_{{\mathfrak {M}}}$ supports the truth of every $B \in \varGamma$, then ${{\mathbf {d}}}_{{\mathfrak {M}}}$ support the truth of *A*, where ${{\mathbf {d}}}_{{\mathfrak {M}}}$ is the unique distinguished state from ${\mathfrak {M}}$, and
($$\models ^{-}_b$$)
for every model ${\mathfrak {M}} \in {\mathfrak {C}}$, if${{\mathbf {d}}}_{{\mathfrak {M}}}$ supports the falsity of every $B \in \varGamma$, then ${{\mathbf {d}}}_{{\mathfrak {M}}}$ support the falsity of *A*, where ${{\mathbf {d}}}_{{\mathfrak {M}}}$ is the unique distinguished state from ${\mathfrak {M}}$, or
($$\models ^{+}_c$$)
for every model ${\mathfrak {M}} \in {\mathfrak {C}}$ and every normal world *w* from ${\mathfrak {M}}$, if *w* supports the truth of every $B \in \varGamma$, then *w* supports the truth of *A*, and
($$\models ^{-}_c$$)
for every model ${\mathfrak {M}} \in {\mathfrak {C}}$ and every normal world *w* from ${\mathfrak {M}}$, if *w* supports the falsity of every $B \in \varGamma$, then *w* supports the falsity of *A*.

### Dimathematism and Strong Dimathematism

We may now introduce dimathematism.

*Dimathematism*^11^   is an informational view: some contradictions are such that their truth is supported by some states from some models. That is, where $\sim$ is negation, there are models, ${\mathfrak {M}}$, states *w* from ${\mathfrak {M}}$, and sentences, propositions (or whatever one takes support-of-truth-bearers and support-of-falsity-bearers to be), *A*, such in ${\mathfrak {M}}$ state *w* supports the truth of both *A* and $\sim\!{A}$. Given that a state supports the falsity of *A* iff it supports the truth of $\sim\!{A}$, this is to say that there are some *A*s, models ${\mathfrak {M}}$, and states *w* from ${\mathfrak {M}}$ such that in ${\mathfrak {M}}$ state *w* supports both the truth and the falsity of *A*.

Dimathematism does not presuppose the existence of conscious beings, and it does not presuppose the existence of agents. In order to avoid misunderstandings, it might be helpful to give some examples of what dimathematism is *not*:

- Dimathematism is not an epistemic view. It does not say that there are sentences *A*, models ${\mathfrak {M}}$, and states *w* from ${\mathfrak {M}}$ such that *A* is both known to be true at *w* and known to be false at *w*. Dimathematism does not make any claims about knowledge.
- Dimathematism is not a doxastic view. It does not say that there are sentences *A*, models ${\mathfrak {M}}$, and states *w* from ${\mathfrak {M}}$ such that *A* is both believed to be true at *w* and believed to be false at *w*. Dimathematism does not make any claims about belief.
- Dimathematism is not a view about some kind of agency. It is not concerned with telling *true* and telling *false* as activities, and it is not a theory about the speech acts of asserting and denying, or about verifying and falsifying as activities.

It is important to highlight that dimathematism is not an epistemic or doxastic view, because in the literature there is some confusion about the understanding of Belnap’s four truth values. Belnap (1977a, p. 47) does write that his four values:

are unabashedly epistemic. According to my instructions, sentences are to be marked with either a **T** or an **F**, a **None** or a **Both**, according as to what the computer has been told; or, with only a slight metaphor, according to what it believes or knows.

but in the republication of Belnap (1977a) in Anderson et al. (1992, p. 521), he inserts an important qualification: “or with only a slight (but dangerous) metaphor, according to what it believes or knows.” Situations represented by an atomic formula being assigned a told value are neither epistemic states nor belief states; for a more comprehensive discussion of this point see Wansing and Belnap (2010). There simply is a fundamental difference between information and the propositional attitudes of knowledge and belief, and I fully agree with Barwise (1989, p. 204), who explains that

Information travels at the speed of logic, genuine knowledge only travels at the speed of cognition and inference.

It is important to emphasize that dimathematism is not a view about some kind of agency because otherwise one might wrongly suppose that dimathematism is trivial. One might think that every sentence *A* is such that someone can both tell *A* *true* and tell *A* *false*, or that for any sentence *A*, one can produce an electronic database that contains both *A* and $\sim\!{A}$, so that the database supports the truth of both *A* and $\sim\!{A}$. Here is where particular quantification over languages eventually comes into play. We may state dimathematism more explicitly as follows:

*Dimathematism* is an informational view: some languages *L* and some contradictory *L*-formulas are such that their truth is supported by some states from some *L*-models. That is, where $\sim$ is negation, there are *L*-models, ${\mathfrak {M}}$, states *w* from ${\mathfrak {M}}$, and *L*-sentences, *A*, such in ${\mathfrak {M}}$ state *w* supports the truth of both *A* and $\sim\!{A}$. Given that a state supports the falsity of *A* iff it supports the truth of $\sim\!{A}$, this is to say that there are some *L*-formulas *A*, *L*-models ${\mathfrak {M}}$, and states *w* from ${\mathfrak {M}}$ such that in ${\mathfrak {M}}$ state *w* supports both the truth and the falsity of *A*.

Even trivialists who believe that every sentence of a natural language such as English is true must admit that there *are* languages *L* such that not each and every *L*-formula *A* is a dimathema. Therefore, even trivialists with respect to natural languages would not be justified in maintaining that dimathematism is a trivial theory.

What is the relationship between dialetheism and dimathematism? Is the latter a variant or a generalization of dialetheism? Is every dialetheia a dimathema? These questions are problematic. A dialetheia is, by definition, a contradiction that is both true and false at the actual world of some model. Dimathematism is, however, not committed to assuming actual worlds. If a state *w* from a model supports the truth (falsity) of a formula *A*, this does not mean that *A* is true (false) in a metaphysical sense, but rather that *w* provides information speaking in favour of *A*’s truth (falsity). Given an atomic formula *A*, information states may not only be silent about *A* but may both “tell *A* true” and and “tell *A* false”.

After making the particular quantification over languages explicit, we may now introduce strong dimathematism.

*Strong dimathematism* is an informational view: some languages *L* and some contradictory *L*-formulas are such that their truth is supported by every state from every *L*-model. That is, where $\sim$ is negation, there are *L*-sentences *A*, such that for every *L*-model ${\mathfrak {M}}$ and every state *w* from ${\mathfrak {M}}$, in ${\mathfrak {M}}$ state *w* supports the truth of both *A* and $\sim\!{A}$. Given that a state supports the falsity of *A* iff it supports the truth of $\sim\!{A}$, this is to say that there are some *L*-formulas *A*, such that every *L*-model ${\mathfrak {M}}$ and state *w* from ${\mathfrak {M}}$ are such that in ${\mathfrak {M}}$ state *w* supports both the truth and the falsity of *A*.

The existence of certain non-trivial contradictory logics demonstrates that not only dimathematism but also strong dimathematism is instantiated, and there are several non-ad hoc non-trivial negation inconsistent logics, for example:

- Various non-trivial inconsistent relevance logics containing Aristotle’s Thesis (Mortensen, 1984; Omori, 2016),
- Abelian group logic and Abelian *l*-group logic (Meyer & Slaney, 1989; Paoli 2001),
- The connexive three-valued conditional logic from Cantwell (2008),
- The non-trivial inconsistent connexive constructive logic **C** from Wansing (2005),
- The non-trivial inconsistent bi-connexive constructive logic **2C** (Wansing, 2016) and the non-trivial inconsistent connexive conditional logics from Wansing and Unterhuber (2019),

but also Arieli and Avron’s (1996, 2000) logics of logical bilattices and two recent logics suggested by Kamide (Kamide, 2017; Omori & Wansing, 2018), in which double-negation behaves as classical, respectively as intuitionistic negation. Also, **LP** expanded by a constant b interpreted as B is a non-trivial contradictory logic as (b$\wedge \sim$ b) is valid. Therefore, on the standard interpretation, second-order **LP** (Hazen & Pelletier, 2018; Priest, 2006) is a contradictory logic as well.

Classifying a logic as negation inconsistent presupposes that there exists a unary operator that indeed qualifies as a negation connective in that logic. We may assume that the notion of negation is non-trivial, so that not any one-place connective whatsoever is a negation operator. In the normal modal logic **Triv** (i.e., **K** + $p \leftrightarrow \Box p)$, the necessity operator $\Box$ seems to be a clear example of a unary connective that fails to be a negation operator. What properties are required to give a unary connective the status of a negation is, however, highly controversial, cf. (Gabbay & Wansing, 1999; Horn & Wansing, 2020; Wansing, 1996, 2001). The unary connective that serves as a negation in **C** and **2C**, for example, does not satisfy the condition of Definition 2.3 in Avron et al. (2018) but does satisfy the minimal condition a negation is required to possess in Marcos (2005). According to the latter criterion, the above listed non-trivial contradictory logics are indeed *negation* inconsistent.

A conception pretty much in line with dimathematism has been outlined in Wansing and Odintsov (2016), which is a contribution to Andreas and Verdée (2016). In his review of Andreas and Verdée (2016), Graham Priest remarks concerning (Wansing & Odintsov, 2016) that

[t]he paper also endorses the thought that the correct approach to paraconsistent logic is to take such a logic to be one of information-preservation. It is not clear to me that this is actually very different form preservation of truth of a certain kind. After all, it is standard enough to cash out information as truth in a certain set of worlds (not necessarily possible worlds).

I do not object; information is often or even standardly cashed out as truth in a set of states, but the information that is preserved by semantical consequence of type ($\models ^{+}_a$), ($\models ^{+}_b$), or ($\models ^{+}_c$) does not preserve truth, but a de-ontologized, non-metaphysical notion of support of truth. The positive information carried by a sentence *A* in an interpretation ${\mathfrak {M}}$ is not represented by $\llbracket A \rrbracket$ = $\{ w \mid A \mbox{ is true in } {{\mathfrak {M}}} \mbox{ at } w\}$ but by

$$\begin{aligned} \llbracket A \rrbracket ^+ = \{ w \mid {{\mathfrak {M}}} \text { supports the truth of } A \text { at } w\}, \end{aligned}$$

where, again, support of truth is not a metaphysical concept but an informational one. Moreover, the negative information carried by a sentence *A* in ${\mathfrak {M}}$, represented by

$$\begin{aligned} \llbracket A \rrbracket ^- = \{ w \mid {{\mathfrak {M}}} \text { supports the falsity of } A \text { at } w\}, \end{aligned}$$

is on a par with the positive information carried by *A* in ${\mathfrak {M}}$. In particular, $\llbracket A \rrbracket ^-$ in general is not the Boolean complement of $\llbracket A \rrbracket ^+$.

According to strong dimathematism, there are languages *L* and *L*-formulas *A* such that for all *L*-models ${\mathfrak {M}}$ with a non-empty set of states *W*, $\llbracket A \rrbracket ^-$ $=$ $\llbracket \sim A \rrbracket ^+$ $=$ $\llbracket A \rrbracket ^+$ $=$ $\llbracket \sim A \rrbracket ^-$ $=$ *W*.

## Turning Off on a Slippery Slope

As already mentioned, Priest (2000) distinguishes three levels of paraconsistency, and Beall and Restall (2006) extend this list of levels to a list of four grades of paraconsistent involvement:^12^

**Fig. 2:**
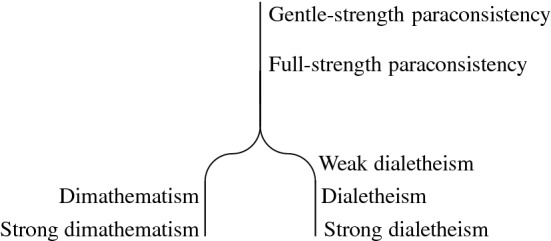
Turning off on a slippery slope

1. Gentle-strength paraconsistency: the rejection of *ex contradictione quodlibet*.
2. Full-strength paraconsistency: the idea that there are interesting or important inconsistent but non-trivial theories.
3. Industrial-strength paraconsistency: the thought that some inconsistent but non-trivial theories are possibly true. (Weak dialetheism in Fig. 2)
4. Dialetheic paraconsistency: the idea that some inconsistent but non-trivial theories are true. (Dialetheism in Fig. 2)^13^

Moreover, Priest (2000) observes that endorsing full-strength paraconsistency comes with a commitment to gentle-strength paraconsistency, but not vice versa, and that industrial-strength paraconsistency requires full-strength paraconsistency, but not conversely. Also, dialetheic paraconsistency implies industrial-strength paraconsistency, but not vice versa, if “possibly true” refers to a notion of possibility such that if a sentence is true, it is also possibly true.

Next, Priest (2000, p. 229 f.) claims that there is a slide from gentle-strength paraconsistency to industrial-strength paraconsistency “forced by the weight of (rational) gravity,” in particular that there is a natural slide from the second level to the third one. He explains that in the semantics of paraconsistent logics, contradictions can be true in a model, and asks why they then cannot be true *simpliciter*. If we understand industrial-strength paraconsistency as expressing weak dialetheism, the question leading to dialetheism is why contradictions cannot be true at the actual world of some model. Priest claims that there is only one move to make, “namely to invoke the Law of Non-Contradiction (LNC) in the form: no contradiction can be true.” But, according to Priest, unless there are convincing arguments for the Law of Non-Contradiction that are acceptable for a defender of full-strength paraconsistency, the slide from the second to the third level of paraconsistency “beckons ineluctably.”

According to Beall and Restall (2006, Sect. 7.4.3), logical pluralism affords an arrest of Priest’s slide. For Beall as a confessing dialetheist, the arrest of Priest’s slide is not without problems, which will, however, not concern us here.^14^ With dimathematism being available, Priest’s slippery slope need not be arrested to be resisted, but rather it can be deflected, see Fig. 2. Dimathematism first of all offers another route to slide down further from full-strength paraconsistency. Moreover, whereas in the case of dialetheism the reason why the slide is not stopped is the lack of convincing arguments for the LNC, dimathematism beckons ineluctably not because of a lack of anything, but because of its plausibility: it is very natural to assume that some information states provide contradictory information and support the truth of contradictions. We have the impression that we are often confronted with contradictory information in different situations. Thirdly, the avoidance of a reliance on a notion of reality may contribute to making dimathematism irresistible.

## Logic’s Non-normativity

It is difficult to exclusively entertain heterodox views, and there is at least one orthodoxy in the philosophy of logic Graham Priest subscribes to. Namely, assuming that “logic is the study of reasoning” (Priest, 1999, Sect. 1.2), Priest holds that logic is normative. In the same paper he explains that “Logic does not tell us how people do reason, but how they ought to reason,” and already in Priest (1979, p. 297) he writes “logic is a normative subject: it is supposed to provide an account of correct reasoning.” The normative status of logic is much debated, for a survey see Steinberger (2017), and it is well-known that the normativity of logic has been challenged by Harman (1984).

I will distinguish between two readings of ‘normative discipline’. According to one reading, a normative discipline has normative consequences insofar as it generates norms of conduct for independent agents that are capable of decision making. These can be norms of morally, culturally, politically, or otherwise correct behaviour. Logic as a normative discipline would then produce norms, i.e., obligations, prohibitions, or permissions, of correct reasoning as an activity or of rational belief formation, supposing that the latter is in an at least indirect sense agentive. Although there is the distinction between ought-to-be and ought-to-do, I assume that norms in the first place apply to agents and not to states of affairs. If it ought to be the case that *A*, for example, this may be understood as there being a norm that obligates some addressees of that norm to see to it that *A*. If a discipline is normative only if it produces prescriptive theories about the conduct of decision making agents, logic cannot be a normative discipline if its laws are descriptive and have nothing to do with reasoning as an activity or with the belief formation of agents.

There exists another reading of the term ‘normative discipline’ according to which a normative discipline generates theories about the meaning of normative notions. In that sense ethics is normative not because it generates norms but because it develops theories about what it means to be obligatory, forbidden, or permitted. Similarly, deontic logic is normative because it develops theories of meaning for operators such as ‘it is obligatory that’. Moreover, the set of normative concepts may be seen to contain expressions such as ‘good’ and ‘bad’ if one assumes that it is forbidden to see to it that something bad is the case and permissible or even obligatory to see to it that something good obtains. Is truth a normative concept? One could maintain that there are intrinsic positive and negative values, and that the good, the bad, truth, rationality, health, or whatever are values in themselves. In that vein, if logic is presented as a discipline that separates good arguments from bad ones, it generates theories about normative concepts, namely the notions of a good argument and a bad argument. If truth is a value, then, on a Fregean understanding of logic, logic is normative in the sense of generating theories about normative concepts because it develops theories about what in general it means to be true.

In this section it will be argued that dimathematism and understanding logic as the study of the most general laws of information flow supports avoiding an entanglement of logic with the normative. Disconnecting logic from the study of reasoning as an activity is a step in that direction. If logic is not about reasoning as an activity at all, it does not have any consequences for how we ought to reason and what we ought to believe.

### Normativity Understood as the Generation of Norms

Disassociating logic from normativity, viewed as the generation of norms, through separating logic form reasoning and from belief formation has been suggested by Shramko (2014). Shramko argues against the normativity of logic by adopting Frege’s anti-psychologistic conception of logic as the science of the most general laws of *being true*. This is, perhaps, surprising because Frege himself believed that logic is a prescriptive normative science. Frege (2013, p. XV) draws the distinction between descriptive and prescriptive laws:

It is commonly granted that the logical laws are guidelines which thought should follow to arrive at the truth; but it is too easily forgotten. The ambiguity of the word “law” here is fatal. In one sense it says what is, in the other it prescribes what ought to be. Only in the latter sense can the logical laws be called laws of thought, in so far as they legislate how one ought to think.

He immediately continues to explain that every descriptive law can be understood prescriptively, but that the descriptive laws of geometry and physics, even if read prescriptively, are different from logic’s prescriptive laws of thought. If the laws of logic were prescriptive in the sense in which the descriptive laws of geometry and physics can be conceived as prescriptive, they would, according to Frege, be psychological laws. They would describe how we actually think. That description could be more or less accurate, and it could be postulated that one should think in accordance with it, but this does not mean that logic itself would generate any prescriptions how to reason. As a part of psychology, logic would not “legislate how one ought to think.”

Shramko (2014, p. 120) distinguishes between three anti-realistic approaches to understanding logical rules, a psychologistic, a linguistic, and a transcendental strategy. According to the psychologistic approach, logical rules “reflect the process of sound human thinking.” Although the notion of reflecting has a descriptive ring, Shramko nevertheless understands the psychologistic approach as a strategy that views logical laws as prescribing how to think correctly. The linguistic approach treats the laws of logic as representing “certain regularities which correspond to structural features of a given linguistic system,” whereas according to the transcendental approach, logical rules “represent fundamental *a priori* structures of consciousness by means of which concepts and intuitions are synthesized to acquire knowledge of the world as it is given in the process of apperception.” What these three approaches have in common is that they restrict “the subject-matter of logic to reasoning, thought or language structures conceived as a kind of human activity” (Shramko, 2014, p. 123). According to Shramko, this “anthropologization” is fallacious because logical laws would continue to exist if mankind ceases to exist. He suggests to define logic as the science of abstract logical entities, namely truth values and truth value functions, whereby logic is an *a priori* rather than an empirical science. As the study of logical entities, logic receives an ontological basis.

The reconciliation of Frege’s view that logical laws are not “psychological laws of taking to be true, but laws of being true” (Frege, 2013, p. XVI) with the idea that logic is a prescriptive normative discipline is an interesting topic that deserves a separate treatment, cf. also Mezzadri (2015). What is important for us is that Shramko takes Frege’s anti-psychologism to arrive at logic as an ontological discipline and to thereby distance logic from the normative. As an ontological discipline, logic is not a normative subject in the sense of generating norms of conduct. Similarly, the understanding of logic as the study of information flow, that underlies dimathematism, separates logic from being a normative discipline in the sense of generating norms. Information flows independently of any decision making agents or belief forming doxastic subjects. Shramko’s de-anthropologization of logic does, however, not show that logic fails to be normative in the sense of studying the meaning of normative concepts.

### Normativity Understood as the Study of the Meaning of Normative Notions

If logic is seen as the study of truth values and truth value functions, one may wonder whether the notions of a truth value and a truth value function are in any sense normative. Although Frege introduced these notions as mathematical concepts, he was influenced by a value-theoretic Neo-Kantian tradition. For example, Frege (1918) takes up Wilhelm Windelband’s triad of basic philosophical values, *true*, *good*, and *beautiful*, when he explains that “just as the word ‘beautiful’ points the way for aesthetics and ‘good’ for ethics, so does ‘true’ for logic” [English translation cited after Gabriel (1984)]. For Frege (1918) truth is what a rigorous science is striving at, and as such, truth might be considered to be an intrinsic value. Thereby also falsity, classically understood as as non-truth, would emerge as a normative concept.

Russell (2020) argues that the laws of logic are not categorically different from the laws of physics or mathematics and that logic “is only entangled with the normative in $\ldots$ a way that it shares with arithmetic and physics, and which does not require logic itself to be normative at all.” She distinguishes between three degrees of normative entanglement a discipline may have. If logic were entangled with the normative to the first degree, it would be normative by definition, and if it were entangled with the normative to the second degree, logic would have normative consequences, which I take to mean that logic would generate norms. Being entangled with the normative only to the third degree, logic has normative consequences “only alongside other (perhaps quite prevalent) normative assumptions.” Defining logic as a normative discipline begs the question, and declaring logic to be entangled with the normative only to the third degree does not answer the question whether logic in general (and not just deontic logic) is normative in the sense of studying the meaning of normative notions.

Russell (2020, footnote 15) remarks that a reviewer pointed out to her that “[l]ogic studies truth, and perhaps truth itself will turn out to be normative, (while physics’ fields and electrons will not.) In that case logic would be more normatively entangled than physics.” Russell basically agrees with the latter, but emphasizes that she assumes that truth is descriptive rather than normative. She also agrees that truth is something we ought to pursue, but she denies that this makes logic a normative discipline. Adopting dimathematism avoids the question whether truth and falsity are normative concepts. Logic is not the science of the most general laws of being true but of the most general laws of information flow from premises to conclusions. However, one may wonder whether the notions of support of truth and support of falsity as informational notions are normative concepts.

They are not, but this is not obvious. Information flow might be truth-preserving if information is understood as being factive (or truthful) as in Floridi’s (2015) theory of “semantic information.” This conception, however, faces the problem that we also speak about misinformation and disinformation. Dunn (2001, p. 423) explains that he likes to

think of information, at least as a first approximation as what is left from knowledge when you subtract, justification, truth, and belief. It is as it were an idle thought. Anyone who has searched for information on the Web does not have to have this concept drummed home. So much of what we find on the Web has no truth or justification, and one would have to be a fool to believe it. It is something like a Fregean “thought,” i.e., the “content” of a belief that is equally shared by a doubt, a concern, a wish, etc. It might be helpful to say that it is what philosophers call a “proposition,” but that term itself would need explanation.

As a Fregean proposition, a piece of information is semantic information, though not in the sense of Floridi’s theory of semantic information. While one may wonder whether the truth of a sentence *A* at a state *w* from a model ${\mathfrak {M}}$ is a value in itself, the support of truth of *A* at *w* form a model ${\mathfrak {M}}$ is not an intrinsic value. The former means that what *A* expresses is indeed the case at *w* in ${\mathfrak {M}}$; the latter only means that *w* speaks in favour of *A* being true at *w* in ${\mathfrak {M}}$. What the sentence ‘state *w* supports the truth of *A*’ expresses may itself be a useful piece of information or not, but if the state *w* supports the truth of *A*, it may then nevertheless be the case that *A* is not true at *w* in a metaphysical sense of being the case or corresponding to the facts. If a state *w* supports the truth of *A* and *w* fails to support the falsity of *A*, this can be stated by saying that *A* receives the value T at *w*. The four cases distinguished between by the four *values* N, F, T, B are on a par, and the statement that a sentence *A* receives one of these values at a state is merely descriptive and if true this does not amount to instantiating or having any positive or negative value. Dimathematism is a view that is helpful in rejecting the claim that logic is a normative discipline in both of the senses distinguished above.

## Summary

In this paper, I have discussed dialetheism as presented by its co-founder and most prominent protagonist, Graham Priest. A distinction has been drawn between various versions of dialetheism as a metaphysical view. If dialetheism is not spelled out in terms of truth and falsity simpliciter but with the help of some elementary notions from possible worlds semantics,

- Single model dialetheism holds that there exist contradictions that are true at the unique actual world @ of the unique actual model,
- Multiple models dialetheism maintains that there exist contradictions that are true at the unique actual world @ of those models that have @ among their worlds,
- Dialetheism claims that there exist contradictions that are true at *some* model’s actual world, and
- Strong dialetheism then is the view that there exist contradictions that are true at *every* model’s actual world.

By contrast, what I have called ‘dimathematism’ is an informational view. It does neither use the concept of a unique actual world, nor does it assume a multiplicity of actual worlds. It replaces the metaphysical notions of truth and falsity at a world by the informational notions of support of truth and support of falsity at an information state.

- Dimathematism holds that there are contradictions the truth of which is supported by *some* states from *some* models, and
- Strong dimathematism maintains that there exist contradictions such that their truth is supported by *every* state from *every* model.

With the availability of dimathematism, rejecting *ex contradictione quodlibet* (gentle-strength paraconsistency) or accepting that there exist interesting or important non-trivial inconsistent theories (full-strength paraconsistency) may be seen to create an attraction not towards dialetheism but towards dimathematism. Moreover, since the notions of support of truth and support of falsity are neither doxastic, epistemic, agentive, nor normative in themselves, dimathematism may be seen to speak against the normativity of logic, where logic is understood as the most general science of information flow from premises to conclusions.

## Footnotes

1 Daniel Skurt and I have made comments on this third heresy in Wansing and Skurt (2020), which is a contribution to Baskent and Ferguson (2019), where one can also find a critical reply by Graham Priest.

2 The literature on dialetheism is vast, and although dialetheism has been heavily criticized it, has now found recognition as a respectable doctrine. A general discussion of dialetheism is beyond the scope of the present paper, for a survey see, for example, Priest et al. (2018).

3 See https://www.metaphysicalsociety.org/.

4 I am grateful to an anonymous referee for asking for such a clarification.

5 Note again that for Priest (2005) all major accounts of truth are compatible with dialetheism.

6 The picture suggests that the set of possible worlds of a model is a subset of the set of impossible worlds, so that every possible world is also an impossible world. This was not intended, and whereas it is said that “[s]ome of them are possible; some of them are impossible,” it is not explicitly said that some are both possible and impossible.

7 However, in a discussion at the conference *Services* *to Logic. 50 Years of the Logician’s Liberation League*, Mexico City, 2019, Graham Priest emphasized that the semantics used in Priest (2006) is used to give an account of semantical consequence, whereas dialetheism is not concerned with semantical consequence.

8 In a discussion at the conference *Services* *to Logic. 50 Years of the Logician’s Liberation League*, Mexico City, October 2019, Priest regretted that he used this modal qualification instead of presenting dialetheism as the thought that some inconsistent but non-trivial theories *are * true.

9 Weak paraconsistency avoids conjunction in the object language; *ex contradictione quodlibet* takes the form $\{A , \sim\!{A}\} \models B$.

10 For a criticism of viewing $\le _t$ as a truth ordering on **4** see Shramko and Wansing (2011).

11 The term ‘dialetheism’ was coined by Graham Priest and Richard Routley (later Sylvan) in 1981 (Priest et al., 1989, p. *xx*); the word ‘dimathematism’ has been made up here. It is derived from the ancient Greek word $\mu \acute{\alpha }\theta \eta \mu \alpha$, which means “that which is learned” or “what one gets to know.” Here it is to be understood as “what one has been told,” and the prefix ‘di’ is meant to indicate that an atomic proposition can be *both* told to be true and told to be false (by different information sources or maybe even one and the same source).

12 In Priest et al. (2018), it is added that there are at least these four grades of paraconsistent involvement.

13 According to the above classification, and assuming that single-model dialetheism and multiple-models dialetheism can be neglected because of the semantics presented in Priest (2006), industrial-strength paraconsistency could be understood as expressing weak dialetheism, and dialetheic paraconsistency would amount to dialetheism.

14 Beall is led to drawing a distinction between the weak and the strong endorsement of a consequence relation.
